# Relationship between teachers’ professional identity and career satisfaction among college teachers: role of career calling

**DOI:** 10.3389/fpsyg.2024.1348217

**Published:** 2024-04-10

**Authors:** Jinshun Wu, Saba Ghayas, Aiman Aziz, Adnan Adil, Sadia Niazi

**Affiliations:** ^1^College of Economics and Management, East China Jiaotong University, Nanchang, Jiangxi Province, China; ^2^Department of Psychology, University of Sargodha, Sargodha, Pakistan; ^3^Department of Psychology, GC Women University Sialkot, Sialkot, Pakistan

**Keywords:** professional identity, career calling, career satisfaction, teaching, mediation professional identity, mediation

## Abstract

**Objectives:**

Professional identity has been found an important determinant of career satisfaction of employees working in various fields. Teachers’ professional identity is a relatively less explored domain. Considering the importance of teacher’s career satisfaction, the current study was designed to test the role of career calling in the relationship between teachers’ professional identity and career satisfaction.

**Methods:**

A sample of teachers (*N* = 300) with (*n* = 115) men and (*n* = 185) women was recruited from the colleges of Sargodha City, Pakistan with an age range from 24 to 45 (M = 31.3, SD = 6.4). A purposive sampling technique was employed for data collection. Variables of the present study were operationalized through the Professional Identity Scale, Brief Calling Scale, and Career Satisfaction Scale.

**Results:**

Path analysis revealed that normative identity did not have any direct or indirect effects on career satisfaction and career calling. Whereas affective and efficacy identity demonstrated significant positive direct effects on career calling whereas career calling had significant positive direct effect on career satisfaction. Efficacy identity also had a significant positive direct effect on career satisfaction. Finally, both affective and efficacy identity had significant positive indirect effects on career satisfaction via career calling.

**Conclusion:**

The findings of the current study may help in devising plans to improve the career satisfaction of teachers working in the higher education sector.

## 1 Introduction

Teachers have a profound influence on the advancement of society and in shaping educational institutions. They assist in educating the next generation and prepare them to be active, accountable citizens by providing them with essential knowledge, skills and values. To guarantee the success and well-being of individuals and societies as a whole, it is essential to recognize teachers’ importance and support their career development. The most important factor that ensures the development of teachers’ professional skills is their career satisfaction. Career satisfaction is referred to as the degree to which individuals are satisfied and fulfilled with their selected profession or job (Wong and Liu, 2024). This shows how much a person cares about his or her work and what sense of purpose and meaning he or she has, as well as the degree to which they feel contented in different aspects of their career. Previous research has demonstrated a relationship between teachers’ career satisfaction and their intentions to leave the profession. Teachers who report having a lower level of career satisfaction are also more likely to consider leaving, and vice versa. In addition, career satisfaction of teachers is strongly related to their overall wellbeing and enthusiasm for professional activities along with the quality of education (Sun et al., 2022; Wong and Liu, 2024). Based on the significance of teacher’s career satisfaction, it is crucial to explore and understand the factors that can improve teaching quality and promote positive outcomes in education.

A teacher’s professional identity refers to the teacher’s strong and optimistic attitude toward their work. It is considered as a sense of attachment to the profession as reflected in the desire to retain the profession and a sense of accomplishment (Ou and Gu, 2024). Teachers with a strong sense of professional identity are committed to their work and view teaching as a long-term passion rather than merely a job. The professional identity of teachers is also reflected in their level of career satisfaction as they find a sense of accomplishment in their work. They are intrinsically motivated when they witness the progress, growth, and academic success of their students (Wong and Liu, 2024). Researchers suggested a strong positive relationship between personal identity and career satisfaction. They argue that, in shaping career satisfaction, a strong sense of personal identity is critical for enhancing commitment and engagement (Perrachione et al., 2008).

### 1.1 Theoretical background and hypotheses

The Social Identity Theory (SIT) assumes that people struggle to attain or maintain a positive social identity and this positive identity stems largely from auspicious comparisons that can be made between the in-group and relevant out-groups. SIT postulates that when people feel a strong connection to their work, they are more likely to be satisfied with their careers (Alexander-Albritton and Hill, 2015). Previously it was elucidated that teachers who have a strong sense of their professional identity are more satisfied with their jobs. They are more likely to feel passionate about their job and have a strong interest in their student’s success. They also are more likely to understand their roles and responsibilities better, which can result in greater career satisfaction (Zheng, 2017). According to researchers, a positive professional identity is essential for teachers’ career satisfaction (Oubibi et al., 2022). This can be achieved through professional development initiatives that help teachers to develop their teaching competencies and to better understand their roles and duties. It can also be achieved by providing teachers with the opportunity to think about their teaching style and to network with other teachers who share their beliefs and values. Based on empirical research and theory, the positive relationship between professional identity and career satisfaction has been confirmed. However, the reasons for this relationship have not been fully elucidated. In this study, we will focus on the reasons why professional identity influences teacher’s career satisfaction. Drawing on previous research (Perrachione et al., 2008; Karaolis and Philippou, 2019) in this study, we hypothesize the teacher’s professional identity as significant positive predictor of a teacher’s career satisfaction (H_1_).

Strong professional identity plays a crucial role in career satisfaction but it is also important to note that it is not the only factor. The literature demonstrates career calling as one of the most discussed topics in the field of occupational psychology (Sabanciogullari and Dogan, 2015; Shang et al., 2022; Wong and Liu, 2024) Career calling is considered as a high level of subjective career success, as it provides a deep sense of meaning and purpose in one’s work. People are also more likely to experience their calling as meaningful—meaningful in the sense that it is “seen as particularly important and carries more positive significance” compared to other activities (Duffy et al., 2012). This is important because meaningfulness is a key determinant of happiness, psychological well-being, and contentment. It has a positive effect on employee satisfaction and career growth (Duffy et al., 2012). Similarly, a study revealed that people with a stronger sense of calling in their careers were more likely to report higher levels of job satisfaction and engagement (Rosso et al., 2010). Research consistently links career calling to improved work performance and experience (Ryan and Deci, 2000; Sabanciogullari and Dogan, 2015). Therefore, the relationship between career calling and career satisfaction is supported empirically and theoretically. Based on the above-cited literature, it is reasonable to propose a hypothesis suggesting career calling as a significant positive predictor of career satisfaction among teachers (H_2_).

Teachers who see their jobs as a calling are more dedicated to their careers and are more likely to find them fulfilling. Career calling is the deepest and strongest pathway for bringing meaningfulness into work. Career calling is a vibrant concept that varies over time and can be molded by antecedent factors, i.e., job involvement (Gazica and Spector, 2015), career pursuit (Dobrow Riza and Heller, 2015), job crafting (Kim et al., 2018), career commitment, and satisfaction (Shang et al., 2022). The literature on career calling has underscored its significance as a predictor. The modern view describes career calling as stemming from within an individual and can also be affected by professional identity. Strong professional identities are associated with more pride in one’s work, better levels of devotion to one’s career objectives, and ultimate advancement in one’s field and self-realization. Conceptually, professional identity and career calling are tightly intertwined. Both concepts highlight a passionate dedication to a job and tend to signify consistency toward a career. They highlight how people describe themselves in the context of their work. However, those who have a strong sense of calling often see their work as a source of meaning and purpose (purposefulness or meaningfulness), and they utilize it to help others or for the greater good. The direction of causality in the relationship between professional identity and career calling is supported by empirical data (Zhang and Guo, 2023). In a sample of medical students 2 years later, it was discovered that professional identity was a strong predictor of career calling (Shang et al., 2022). Similar to this, results of a longitudinal study revealed that people with distinct professional identities are more likely to discern their career calling over time. Consequently, professional identity may serve as a requirement for the emergence of calling (Duffy et al., 2012). Thus, it is hypothesized that professional identity will be a positive predictor of career calling among teachers (H_3_).

For teachers, previous research has established somewhat consistent evidence of a relationship between professional identity, career calling, and career satisfaction. We isolate career calling as a linking mechanism between professional identity and career satisfaction. It is becoming more common to refer to career calling as a secular concept that emphasizes self-personal fulfillment, actualization, and passion. Calling reflects an active involvement in one’s vocational development (Dik and Shimizu, 2019). Professional identity is defined as quite a stable and lasting group of attributes, values, beliefs, experiences, and motives in terms of which people delineate themselves in a professional role (Praskova et al., 2015). Strong professional identities are associated with greater pride in one’s work, higher levels of commitment to one’s career goals, and eventual advancement through one’s field and self-realization (Li et al., 2015; Jue and Ha, 2018). The relationship between professional identity and career calling has been conventional in empirical research. According to the “onion model of a good teacher,” a teacher’s professional identity, or what it means to be a teacher, has a significant impact on their mission, or what they perceive to be their calling in the world (Korthagen, 2013). This viewpoint is in line with the theoretical idea that one’s calling and identity are inextricably linked (Pratt et al., 2003). The above cited literature provides support to the direction of the relationship between professional identity and career calling. Both concepts emphasize a passionate commitment to a career and tend to signify constancy toward career satisfaction. The possibility of having a career calling, which in turn has a beneficial influence on career satisfaction, is specifically affected by professional identity (Hirschi and Herrmann, 2013). As reviewed above, both professional identity and career calling are positively linked to career satisfaction. Additionally, research has revealed a positive relationship between professional identity and career calling. This leads to the question of whether the relationship between professional identity and career satisfaction is sequentially mediated by career calling. If possible then a pathway that professional identifies influences career satisfaction via career calling should be taken into account. Therefore, it is suggested that career calling may mediate the relationship between professional identity and career satisfaction (H_4_).

In conclusion, it is crucial to understand how a teacher’s professional identity and sense of calling are related because this has an impact on how engaged, contented, and committed they are to their work as teachers. A stronger sense of professional identity, intrinsic motivation, and a sense of purpose and meaning in one’s work are all more likely in teachers who see their work as a calling in life. Therefore, it is necessary to conduct more research to determine the factors that influence career calling in teachers and how it affects the teaching profession. Pakistani researchers underscore the significance of understanding teacher identity formation within the context of Pakistan, highlighting it as a foundational stage in a teacher’s professional journey. They argue that while academics have extensively explored teacher identity globally, there is been limited attention to this concept specifically in Pakistan. This lack of focus is concerning given the unique cultural and social dynamics shaping the teaching profession in the country. Studies suggest that teaching is often perceived as a less esteemed profession, chosen mainly by females due to cultural expectations or financial constraints rather than genuine passion. However, existing research on teacher identity primarily focuses on those already in the profession or at the tertiary level, neglecting the crucial phase of pre-service training. Nawab and Khanzada emphasize the need to understand how teachers negotiate their identity during pre-service training, as this stage lays the groundwork for their professional journey. They argue that tailored research in this area is essential to inform the design of teacher education programs in Pakistan, ensuring they effectively support the development of teacher identity and ultimately improve the quality of education in the country (Nawab and Zada, 2023).

## 2 Methods

### 2.1 Sample

A purposive sample (*N* = 300) was drawn from College teachers of Sargodha city, Pakistan. Men (*n* = 115) and women were given (*n* = 185) represented in the sample. Teachers from private and government colleges were included in the study. The age range of the sample was 24–45 (M = 31.3, SD = 6.4).

### 2.2 Instruments

#### 2.2.1 Teachers professional identity

This scale consists of 15 items, for which students rated on a four-point Likert self-report scale (1 = strongly disagree, 2 = disagree, 3 = agree, and 4 = strongly agree). It has three subscales. Affectional Identity consists of six items (I like teaching) with Cronbach’s α = 0.88, of Normative Identity consists of five items (I often actively participate in training and lectures for teachers and teaching for promotion) with Cronbach’s α = 0.78 and Efficacy Identity consists of four items (Teacher is a highly respected occupation) with Cronbach’s α = 0.75. Items 1 and 13 are reverse coded. The whole scale showed acceptable reliability, Cronbach α = 0.78, as did each dimension (Cronbach α = 0.88, 0.78, and 0.75, respectively; Zhang et al., 2016).

#### 2.2.2 Career calling

Career Calling was assessed by using the Brief Calling Scale (Dobrow and Tosti-Kharas, 2012) The scale consists of 12 items (e.g., “I would sacrifice everything to be a teacher” or “I would continue being a teacher even in the face of severe obstacles”). Items were rated on a five-point Likert scale (1 = strongly disagree and 5 = strongly agree). Higher mean scores show a stronger career calling; the Cronbach’s alpha coefficient for this scale in the present study was 0.95.

#### 2.2.3 Career satisfaction

Career satisfaction was measured through the Career Satisfaction Scale (Greenhaus et al., 1990). The scale comprises of five items (I am satisfied with the success, I have achieved in my career). Response format of the scale is five-point Likert scale. The Cronbach’s alpha coefficient for this scale in the present study was 0.75.

### 2.3 Procedure

Before data collection topic of the study was approved by Intituitional Review board. After ensuring the suitability of the instruments for the current study, a sampling plan was checked out to ensure the representativeness of the sample. After that, data were collected by college teachers Sargodha. After obtaining permission, individuals were personally approached. Informed consent included the reason for the study, procedure, and their rights as a contributor, as well as the confidentiality of the information that was guaranteed to the participants. The participants were briefed approximately the goals and reason of the observation. Rapport was established and members were assured that confidentiality of their private data could be maintained and results were used for research purposes very carefully. For data collection, every member was approached personally, and their information was not shared with others and written consent was obtained from all individuals. Clear instructions were given to all members about the instruments and response method, written questionnaires were provided to the members, and questionnaires were marked by them accordingly. Data collection turned to be completed in 2 months. The obtained results were further processed to statistical evaluation.

## 3 Results

In the present study, impact of teacher’s professional identity on career satisfaction through career calling was examined. The hypotheses were tested through path analysis whereas psychometric properties were ensured and descriptive statistics was also performed.

Table 1 shows Cronbach’s alpha coefficients of reliability, and interscale correlations for the focal constructs of the present study. The Cronbach’s alpha coefficients of reliability for all the scales were > 0.70 indicating satisfactory degree of internal consistency of the measures. The correlation matrix suggested that all variables were significantly and positively correlated with one another.

**Table 1:** Descriptive statistics and intercorrelations of the focal variables (*N* = 300)

Variables	*M*	*SD*	Range		*Sk*	1	2	3	4	5
			Actual	Potential						
1.Affective identity	19.79	2.75	8–24	6–24	−0.85	-	0.75^*^	0.51^*^	0.51^*^	0.31^*^
2.Normative identity	9.26	1.50	3–12	5–20	−0.72	-	-	0.62^*^	0.45^*^	0.38^*^
3.Efficacy identity	17.82	3.14	9–14	4–16	−0.32	-	-	-	0.58^*^	0.48^*^
4.Career calling	42.03	8.29	20–60	5–120	−0.15	-	-	-	-	0.59^*^
5.Career satisfaction	16.82	4.24	5–25	5–25	0.02	-	-	-	-	-

**Figure 1:**
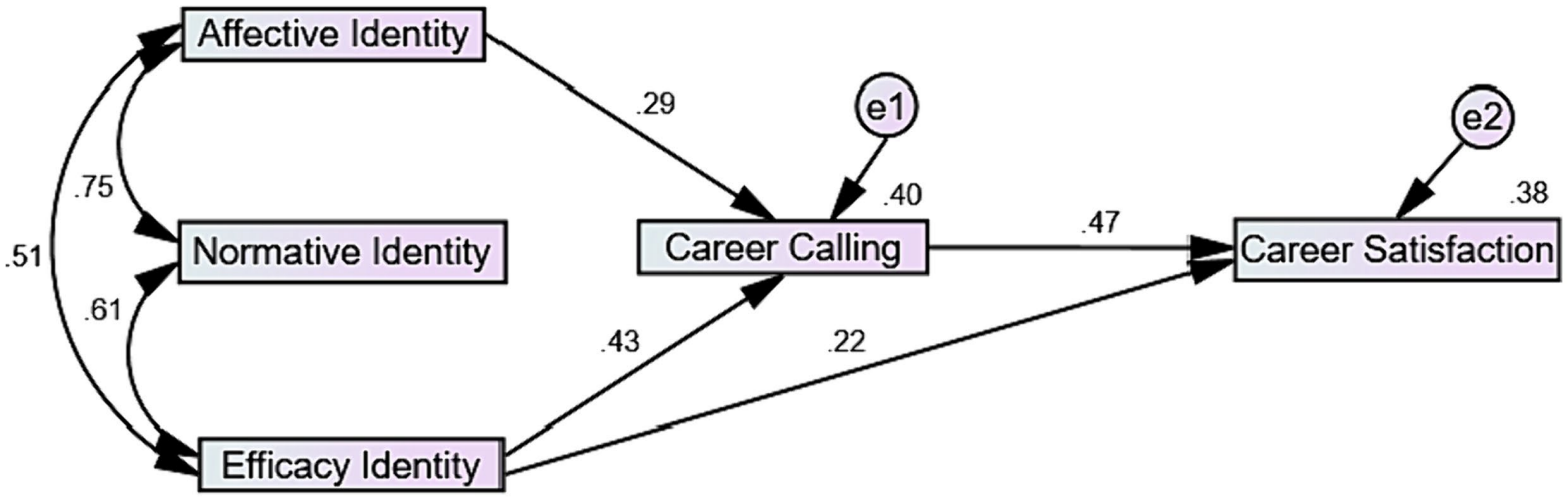
Path diagram of the proposed model of the present study.

Table 2 presents the standardized path coefficients of direct and indirect effects computed through maximum likelihood estimation along with 95% bias corrected confidence intervals across 5,000 bootstrapped samples. The model fitted well to the data as the χ^2^ = 4.18, *df* = 3, *p* = 0.24. All other fit indices also indicated a very good fit to the data (CFI = 0.99, GFI = 0.99, Standardized RMR = 0.014, RMSEA = 0.047 with *p*_close_ = 0.42). Normative identity did not have any direct or indirect effects on any of the focal variables. Affective and efficacy identity demonstrated significant positive direct effects on career calling whereas career calling had significant positive direct effect of career satisfaction. Efficacy identity also had a significant positive direct effect on career satisfaction. Finally, both affective and efficacy identity had significant positive indirect effects on career satisfaction via career calling. Figure 1 depicts the path diagram of the model of the present study.

**Table 2:** Standardized path coefficients of direct and indirect effects (*N* = 300).

Paths	*β*	*p*	95% CI for *β*	
			LL	UL
Affective Identity → Career Calling	0.29	0.001	0.14	0.46
Efficacy Identity → Career Calling	0.43	0.001	0.28	0.56
Efficacy Identity → Career Satisfaction	0.22	0.005	0.07	0.36
Career Calling → Career Satisfaction	0.47	0.001	0.31	0.62
Affective Identity → Career Calling → Career Satisfaction	0.14	0.001	0.07	0.24
Efficacy Identity → Career Calling → Career Satisfaction	0.20	0.001	0.12	0.31

## 4 Discussion

The current study was aimed at exploring the relationship between professional identity and career satisfaction among college teachers. Furthermore, it was also intended to explore the mediating role of career calling in this relationship. The hypothesis of the study was aimed at exploring the mediating role of career calling in the relationship between professional identity and career satisfaction. When this hypothesis was tested through SEM, it was found that normative identity did not have any direct or indirect effects on any of the focal variables. Normative identity refers to the perception of an individual about the societal norms and expectations surrounding their chosen career or profession. While it is a critical aspect of identity formation, it may not directly influence career satisfaction for certain professional groups, such as teachers. Teachers are known for their intrinsic motivation and dedication to their profession, driven by a deep sense of commitment to educating and shaping young minds. This vocational calling is rooted in their passion for teaching and the genuine desire to make a positive impact on students’ lives. Consequently, the influence of normative identity, which focuses on societal norms, may be overshadowed by teachers’ strong vocational values. Also that the career satisfaction of teachers is often influenced by factors unique to the education profession, such as the quality of student-teacher interactions (Greenhaus et al., 1990; Dobrow and Tosti-Kharas, 2012; Zhang et al., 2016; Zheng, 2017; Karaolis and Philippou, 2019), the sense of accomplishment from helping students succeed, appreciation, acknowledgement, and respect from students (Lee et al., 2022), and the fulfillment derived from fostering intellectual growth. As normative identity typically centers on broader societal expectations, it might not encompass the intricacies and nuances of career satisfaction within the teaching profession.

Teachers often find intrinsic motivation and autonomy within their role (Ou and Gu, 2024), which can significantly contribute to their job satisfaction. When educators derive satisfaction from fulfilling their intrinsic desires and self-determined goals, the influence of external factors like normative identity becomes less pronounced. It is possible that other dimensions of identity, such as affective identity (emotional attachment to the teaching profession) and efficacy identity (belief in their teaching abilities), may play more prominent roles in shaping career calling and career satisfaction among teachers. These dimensions could overshadow the impact of normative identity within the context of our study.

Results also showed that affective and efficacy identity demonstrated significant positive direct effects on career calling whereas career calling had a significant positive direct effect of career satisfaction. Affective identity refers to the emotional attachment an individual has toward their chosen career or profession (Kim et al., 2018). Teachers with a strong affective identity may feel a deep sense of purpose and fulfillment in their roles, viewing teaching not merely as a job but as a vocation. This emotional attachment can lead them to perceive their teaching career as a calling—an inherent inclination to engage in work that holds personal significance and a sense of higher purpose as supported by researchers (Duffy et al., 2012) wherein they found career calling to be positively related to multiple variables making professional identity one of those variables. Whereas efficacy identity pertains to an individual’s belief in their abilities and competence related to their profession (Perrachione et al., 2008). A significant positive relationship between efficacy identity and career calling could be because teachers with high efficacy identity are likely to perceive themselves as capable and skilled educators, confident in their abilities to make a positive impact on their students’ lives. This self-assurance and belief in their teaching prowess can reinforce their perception of teaching as a calling, as they feel equipped to excel in their profession and make meaningful contributions.

Career calling involves a sense of purpose and passion for one’s profession, as well as the belief that the work aligns with one’s values and identity. When teachers experience a strong sense of calling in their profession, they are more likely to find intrinsic motivation, fulfillment, and meaningfulness in their work. This, in turn, contributes to higher levels of career satisfaction as researched in broader scholarly work, calling was found to be an associated factor with multiple variables along with career satisfaction (Zhao et al., 2012). Feeling a calling in their teaching profession allows teachers to derive a sense of meaning and purpose from their work, leading to increased job satisfaction and overall well-being and it builds a strong relationship between calling and work satisfaction. Important studies have revealed a connection between career calling and work effort, dedication, and wellbeing (Dik and Shimizu, 2019). Duffy and colleagues also found a strong relationship between calling and job satisfaction where career commitment served as a mediator (Sabanciogullari and Dogan, 2015).

In the same analysis, efficacy identity was also seen to have a significant positive direct effect on career satisfaction. This finding suggests that teachers who possess a strong belief in their teaching abilities and competence (efficacy identity) are more likely to experience higher levels of career satisfaction. Efficacy Identity is associated with an individual’s perception of their capabilities and effectiveness in their role or profession. When individuals have a strong efficacy identity as teachers, they tend to believe in their abilities to handle challenges, meet the demands of their job, and achieve positive outcomes in their teaching endeavors. This heightened confidence positively affects their performance, leading to successful teaching experiences and increased job satisfaction. According to researchers teachers’ skills and abilities is one of the factors that contribute in their career satisfaction (Greenhaus et al., 1990). In a study, researchers revealed self-efficacy positively affects compensation, wage growth, and career satisfaction at the entry level of a career. A strong sense of efficacy identity fosters a proactive and motivated approach to work (Betoret, 2006). Teachers who believe in their capabilities are more likely to tackle their responsibilities with enthusiasm and persist in overcoming obstacles through crafting their jobs. This intrinsic motivation and authority to craft their job as per demands and needs can enhance their dedication and investment in their teaching profession, resulting in higher levels of career satisfaction (Jepson and Forrest, 2006).

Another plausible explanation is that teachers who possess an efficacy identity are better equipped to handle the challenges and stressors that may arise in their profession. Their belief in their problem-solving skills and abilities to adapt to changing circumstances enhances their resilience. This ability to cope with difficulties positively impacts their overall well-being and career satisfaction (Hargreaves, 2000; Spilt et al., 2011). They added that people with high levels of resilience reported better health, self-esteem, and work control during difficult times, all of which are associated with greater effectiveness and higher productivity. Furthermore, teachers with a strong efficacy identity are more likely to feel a sense of commitment and loyalty to their educational institution. This commitment can lead to a deeper connection with the organization and its goals, which, in turn, enhances career satisfaction (Retelsdorf et al., 2010).

Finally, the same analysis found that both affective and efficacy identity had significant positive indirect effects on career satisfaction mediated by career calling. Since both affective identity and efficacy identity are positively associated with career calling, teachers with a strong sense of affective identity are more likely to perceive their work as a calling. People who are living their calling experience intense levels of fulfillment in their careers and personal lives (Chapman and Lowther, 1982; Xie et al., 2016). As career calling is associated with increased career satisfaction, this sense of calling acts as a mediating factor between affective identity and career satisfaction. In this study, career calling acts as a mediator between affective and efficacy identity and career satisfaction. When teachers view their work as a calling, it becomes the bridge through which their emotional connection and belief in their teaching abilities impact their overall career satisfaction. It was supported and found that Korean hotel employees’ sense of calling has a significant positive relationship with career satisfaction (Abele and Spurk, 2009; Dubbelt et al., 2019).

When people approach their jobs with a calling perspective, their identities and professions become interwoven, they view their work as genuinely joyful, and they think that it contributes significantly to society (Haar and Staniland, 2016; Srivastava and Madan, 2020). Let us consider a passionate and effective teacher who deeply cares about their students and strongly believes in their teaching abilities. They find great joy and fulfillment in their role as an educator (high affective and efficacy identity). These emotions and beliefs create a strong sense of purpose and meaning in their work. They perceive teaching not just as a job but as something significant and personally fulfilling—a calling.

Now, this perception of teaching as a calling influences their overall career satisfaction. Because they feel a strong sense of purpose and meaning in their work, they derive higher levels of satisfaction from their profession. The emotional connection, passion, and belief in their abilities enhance their career satisfaction through the sense of calling they experience. A similar mediating relationship has been shown in a study by Dobrow and his colleagues wherein he found that that people with a stronger sense of calling in their careers were more likely to report higher levels of job satisfaction and engagement (Hall and Chandler, 2005; Yap et al., 2010; Duffy et al., 2012). Hence, when people are motivated internally, as opposed to externally, they work with greater interest, excitement, and confidence, and when they engage in self-motivated behavior, their vitality, self-esteem, and overall wellbeing may increase (Ryan and Deci, 2000; Steger and Dik, 2009; Chen et al., 2023; Ou and Gu, 2024).

## 5 Conclusion

The current study was aimed at exploring the relationship between professional identity and career satisfaction among college teachers. Furthermore, it was also intended to explore the mediating role of career calling on the relationship between professional identity and career satisfaction among college teachers. The findings of the study revealed that all the study variables had significant positive correlations with each other and had good and excellent reliabilities. Through SEM, it was found that affective and efficacy identity demonstrated significant positive direct effects on career calling whereas career calling had a significant positive direct effect on career satisfaction. Efficacy identity also had a significant positive direct effect on career satisfaction. Finally, both affective and efficacy identity had significant positive indirect effects on career satisfaction via career calling. Mediation analyses revealed that professional identity, i.e., affective identity, and efficacy identity had significant positive direct and indirect effects on career satisfaction. Mediation analysis also revealed that career calling had a significant positive relationship with career satisfaction and career calling among college teachers.

### 5.1 Limitations and suggestions of the study

While our study employed rigorous methodologies and robust statistical analyses, it is essential to acknowledge the limitations inherent in any research. The study used a cross-sectional approach, which only offers a momentary picture of the associations. Experimental or longitudinal designs might provide more information about the causes of the connections between the variables.

The sample of college professors could not accurately reflect the range of experiences and backgrounds among college teachers. The results might not apply to all college instructors, especially those from other regions or educational environments. Self-report measures were used to gather the data, which makes them vulnerable to social desirability bias and common method bias.

When examining the relationship between professional identity and career satisfaction, the study looked at the mediating roles of career calling. A mediation analysis does not prove causation, and other unmeasured factors may be also having an impact on the connections. It is suggested to utilize cutting-edge statistical methods, such as causal mediation analysis or bootstrapping, to more thoroughly investigate.

### 5.2 Implications

The implications of the current study findings can have significant implications for the field of education and the well-being of college teachers. Here are some potential implications:

1. The creation of more efficient teacher preparation programs can be influenced by an understanding of the connections among professional identity, career satisfaction, and career calling of college instructors. Teacher training programs can better prepare educators for rewarding and gratifying careers by building a strong professional identity, a feeling of career calling, and satisfaction.
2. The study’s findings can provide insight into the elements that influence college teachers’ overall career satisfaction and job fulfillment. By addressing issues linked to professional identity and career calling, institutions and policymakers can use this knowledge to adopt strategies and programs that improve teacher retention rates.
3. When educational institutions take into account the career calling’s mediating roles in the relationship between professional identity and career satisfaction, support structures for teachers’ sense of autonomy, competence, and purpose can be put in place. Giving teachers chances for professional development and progress can have a good effect on their sense of job satisfaction and drive to do well in their positions.
4. For college teachers, having a better understanding of the variables affecting career satisfaction might result in a better work-life balance. To lessen burnout and improve overall wellbeing among educators, supportive work environments that emphasize professional identity, career calling, and psychological empowerment can be created.
5. The conclusions can be used to direct the creation and execution of programs for professional development for teachers. These programs can be made to better teachers’ career satisfaction and increase the effectiveness of their instruction by addressing the elements of professional identity and career calling.
6. Teachers who are satisfied with their careers are more likely to be motivated and successful in their teaching strategies, which can have a favorable effect on student learning results. Through the happiness and satisfaction of their teachers, the current study’s findings indirectly improve students’ educational experiences.

## Data availability statement

The raw data supporting the conclusions of this article will be made available by the authors, without undue reservation.

## Ethics statement

The studies involving humans were approved by Institutional review board, University of Sargodha. The studies were conducted in accordance with the local legislation and institutional requirements. The participants provided their written informed consent to participate in this study.

## Author contributions

JW: Data curation, Funding acquisition, Methodology, Supervision, Writing – review & editing. SG: Conceptualization, Investigation, Writing – review & editing. AAz: Formal analysis, Writing – original draft. AAd: Software, Validation, Visualization, Writing – original draft. SN: Resources, Writing – original draft.
